# Microbial Translocation in Chronic Liver Diseases

**DOI:** 10.1155/2012/694629

**Published:** 2012-07-17

**Authors:** Marilia Rita Pinzone, Benedetto Maurizio Celesia, Michele Di Rosa, Bruno Cacopardo, Giuseppe Nunnari

**Affiliations:** ^1^Division of Infectious Diseases, Department of Clinical and Molecular Biomedicine, University of Catania, 95125 Catania, Italy; ^2^Department of Microbiology and Immunology, Jefferson Medical College, Thomas Jefferson University, Philadelphia, PA 19107, USA

## Abstract

The intestinal microflora is not only involved in the digestion of nutrients, but also in local immunity, forming a barrier against pathogenic microorganisms. The derangement of the gut microflora may lead to microbial translocation, defined as the passage of viable microorganisms or bacterial products (i.e., LPS, lipopeptides) from the intestinal lumen to the mesenteric lymph nodes and other extraintestinal sites. The most recent evidence suggests that microbial translocation (MT) may occur not only in cirrhosis, but also in the early stage of several liver diseases, including alcoholic hepatopathy and nonalcoholic fatty liver disease. Different mechanisms, such as small intestinal bacterial overgrowth, increased permeability of intestinal mucosa, and impaired immunity, may favor MT. Furthermore, MT has been implicated in the pathogenesis of the complications of cirrhosis, which are a significant cause of morbidity and mortality in cirrhotic subjects. Therapeutic strategies aiming at modulating the gut microflora and reducing MT have focused on antibiotic-based options, such as selective intestinal decontamination, and nonantibiotic-based options, such as prokinetics and probiotics. In particular, probiotics may represent an attractive strategy, even though the promising results of experimental models and limited clinical studies need to be confirmed in larger randomized trials.

## 1. Introduction

The intestinal microflora is a complex ecosystem, consisting of more than 500 microbial species, that are involved in the digestion of nutrients and the production of vitamins and short-chain fatty acids; furthermore, the gut microflora plays a role in local immunity, forming a barrier against pathogens, together with the intestinal mucosa [1]. The derangement of the gut microflora and increased microbial translocation (MT) have been widely described in advanced liver disease and associated with the pathogenesis of the complications of cirrhosis [2]; moreover, recent lines of evidence suggest the intestinal microflora to be directly implicated in the induction and progression of liver damage in several chronic liver diseases, including alcoholic and non-alcoholic steatohepatitis, two common causes of cirrhosis [3–5].

Here, we review current concepts regarding the pathogenesis of MT, its role in liver diseases and the potentialities of therapeutic strategies based on the modulation of intestinal flora (i.e. probiotics). 

## 2. Microbial Translocation in Cirrhosis

Microbial translocation (MT) is defined as the migration of viable microorganisms or bacterial endotoxins (i.e., bacterial lipopolysaccharide (LPS), peptidoglycan, and lipopeptides) from the intestinal lumen to the mesenteric lymph nodes (MLN) and other extraintestinal sites [6, 7]. Gram-negative members of the Enterobacteraceae family (such as *Escherichia coli* and *Klebsiella *spp.), enterococci, and other streptococci species are the most effective at bacterial translocation to MLN, across even histologically normal intestinal mucosa [8, 9]. On the contrary, anaerobic species only rarely translocate and they have been reported to limit the growth of aerobic species with higher translocation potentialities [10]. 

Increased MT has been described both in experimental animal models of cirrhosis and in cirrhotic patients. In animal studies, the prevalence of MT, defined as a positive bacteriological culture from surgically removed MLN, was around 50% in cirrhotic rats with ascites [11] and up to 80% in cirrhotic rats with spontaneous bacterial peritonitis (SBP) [12–14]. In humans, few studies are available, because of the difficulty in detecting MT to MLN and the lack of widely applicable noninvasive markers of MT. However, MT seems to occur more frequently in Child C cirrhotic patients (about 30% of positive MLN cultures), in comparison with Child B and A cirrhotic patients (8% and 3%, resp.) [15]; in addition, levels of tumour necrosis factor (TNF)-*α* were found to be higher in MLN of cirrhotic patients than in controls and to correlate with Child-Pugh score and to the risk of developing bacterial infections during the first month after transplant [16].

Detection of bacterial deoxyribonucleic acid (bactDNA) in blood and ascites using the polymerase chain reaction (PCR) has been proposed as a sensitive surrogate marker of MT [17–19]. Such et al. [17] detected the presence of bactDNA (mainly *E. coli*) simultaneously in blood and ascites in as many as 32% of patients with advanced cirrhosis and sterile nonneutrocytic ascitic fluid. Of importance, bactDNA sequence similarity in blood and ascites suggested a shared origin, from a common MT event. Unfortunately, there was no correlation between the severity of liver disease and the detection of bactDNA in body fluids, so that the clinical impact of molecular methods to detect MT remains to be established.

### 2.1. Pathogenesis of Microbial Translocation

**Figure 1:**
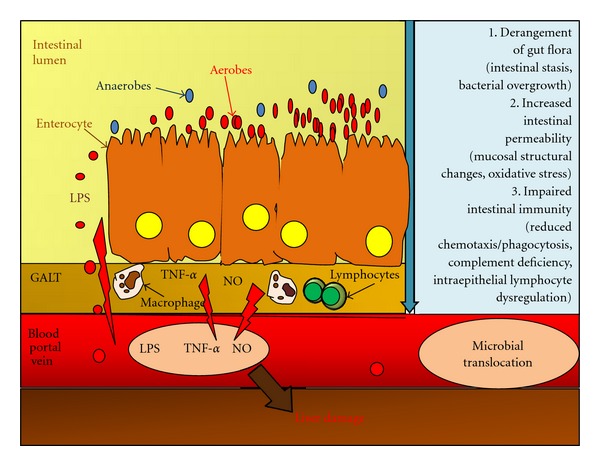
Mechanisms of microbial translocation in chronic liver diseases. LPS: lipopolysaccharide; NO: nitric oxide; GALT: gut-associated lymphatic tissue; TNF-*α*; tumour necrosis factor.

Cirrhosis may lead to MT via different mechanisms, including small intestinal bacterial overgrowth (SIBO), disturbance of luminal factors, increased permeability of intestinal mucosa, and impaired immunity. These factors are summarized in Figure 1.

**SIBO**

SIBO has been shown to frequently occur in the setting of chronic liver diseases and to be related to the degree of hepatic dysfunction [20]. The diagnosis of SIBO is based on the use of glucose breath hydrogen tests [21, 22] or quantitative culture of jejunal aspirates [20, 23]. The limited sensitivity of breath tests [24] may explain the lower prevalence rates of SIBO (about 30–38% *versus* 48–73%) found when using breath tests [21, 22] rather than jejunal colony counts [23, 24] to detect bacterial overgrowth.

A number of explanations may account for SIBO, including hypochlorhydrosis, malnutrition, and intestinal hypo-dysmotility [24–26]. The pathogenesis of small intestinal hypomotility in cirrhosis is multifactorial, as a result of increased adrenergic activity, enhanced nitric oxide (NO) production, and structural intestinal damage, due to oxidative stress and portal hypertension [20, 27]. Of note, SIBO itself may further compromise intestinal motility [28, 29], thus creating a vicious cycle that amplifies bacterial overgrowth. In animals, the administration of prokinetics, like cisapride [23, 30], and *β*-adrenergic blockers, like propanolol [31], has been reported to reduce SIBO and MT; in clinical practice, a six-month trial with cisapride showed decreased orocaecal transit time and SIBO elimination in 80% of patients with SIBO at baseline [23, 32]. Unfortunately, cisapride has been withdrawn from use in some countries because of potential for cardiac arrhythmia.

**Disturbance of Luminal Factors**

Several luminal factors, such as bile acids, secretory immunoglobulin A, mucins, defensins, lysozyme, and phospholipase A2, physiologically contribute to the intestinal barrier against MT. Bile acids inhibit bacterial overgrowth, especially of anaerobic species; moreover, the absence of bile in the intestine has been shown to promote MT [33]. In cirrhosis, the reduced secretion of bile acids may favor SIBO and MT. Of interest, oral administration of conjugated bile acids, cholylsarcosine, and cholylglycine has been reported to reduce intestinal bacterial content and to decrease the rate of MT in ascitic cirrhotic rats [34]. Further studies should evaluate the potential benefits of bile acids in humans.

**Impaired Immunity**

Cirrhosis is accompanied by several abnormalities of both systemic and local immune system. The reticulo-endothelial system (RES), whose activities are mainly located in the liver (Kupffer cells), is the main defense against bacteremia; in cirrhosis, phagocytic activity of Kupffer cells may be impaired and the presence of portosystemic shunts bypassing the liver (thereby escaping the action of the RES) may explain the failure to clear not only portal or systemic bacteria, but also endotoxins and cytokines [35]. In cirrhotic subjects, serum complement levels have been reported to be low [36, 37] and to independently predict the risk of infections and mortality [37]. Cirrhosis is also accompanied by a decrease in bactericidal activity by monocytes and neutrophils [38, 39]. An increased number of intraepithelial lymphocytes with markedly impaired proliferative activity and capacity to produce interferon-*γ* have been reported in a murine model of cirrhosis and correlated with increased MT [40]. Considering the crucial role of intestinal immune cells in regulating the interplay between the host and gut flora, it can be assumed that the derangement of local immunity may allow translocated bacteria to escape from MLN and to reach systemic blood and other extraintestinal sites.

**Increased Permeability**

Structural and functional changes in intestinal mucosa may increase its permeability, contributing to the development of MT. Structural abnormalities, whose most important determinant appears to be portal hypertension, include vascular congestion, thickened muscularis mucosa, fibromuscular proliferation, and a reduced villus/crypt ratio [41]. In addition, ultrastructural abnormalities in the epithelial layer of small intestine specimens of cirrhotic subjects have been described [42]. In experimental cirrhosis, oxidative damage of the intestinal mucosa has been shown to cause lipid peroxidation of the brush border membranes and abnormal intestinal transport [43, 44]. Most functional studies, usually dual sugar absorption tests, have found increased intestinal permeability in patients with advanced liver disease [45–48]. Increased permeability is likely to occur via a paracellular route. In humans, morphologically intact tight junctions at the apical pole of enterocytes have been reported in a cohort of clinically stable cirrhotic patients with no prior history of infection with gut-derived bacteria [42], but it remains to be established whether tight junctions are intact in cirrhotic patients with a history of infection with enteric bacteria. Notably, dilatation of the intercellular space below tight junctions has been documented in patients with cirrhosis [32] and, more importantly, NO, whose role in cirrhosis is discussed below, has been shown to reversibly dilate tight junctions, to destroy the cytoskeleton, and to inhibit the formation of adenosine triphosphate in cultured intestinal epithelial cells [49].

### 2.2. Not Only Microbes: Endotoxins and Cytokines in Cirrhosis

As previously stated, MT does not only refer to the passage of viable bacteria, but also of microbial products, across the gut mucosa, thus causing the production of proinflammatory cytokines and vasoactive mediators. Patients with cirrhosis have been reported to have increased circulating levels of endotoxin (LPS), a cell wall component of Gram-negative bacteria [50, 51]; MT, as well as reduced hepatic clearance, may be responsible for the spill-over of intestinal endotoxin into the systemic circulation. In fact, endotoxemia is significantly more frequent in cirrhotic rats with MT than in cirrhotic animals without MT [52]. Furthermore, MT itself has been reported to make the gut a “cytokine-releasing organ”, even in the absence of portal or systemic bacteremia [53, 54]. Endotoxin-primed macrophages release high levels of TNF-*α* in experimental models of liver damage [55–57]. In rats, TNF-*α* levels in MLN have been shown to be higher in presence of MT and to correlate with TNF-*α* plasma levels [58]; in humans, as previously reported, elevated levels of TNF-*α* in MLN of cirrhotic subjects have been found and significantly correlated with those detected in the blood [16]. The overproduction of proinflammatory cytokines has been associated with elevated plasma levels of LPS-binding protein (LBP), which is synthesized in the liver in response to endotoxin and promotes the binding of endotoxin to the CD14/Toll-like receptor 4 (TLR4) receptor complex [59]. Based on these observations, endotoxemia has been proposed as a major cause of the proinflammatory state in cirrhotic subjects [60–62]. However, other studies have failed to show a significant association between circulating endotoxin and proinflammatory cytokines levels [63–65]. Moreover, contrary to previous assumptions, Riordan et al. suggested to focus more on Gram-positive bacteria rather than Gram-negative ones: in fact, the authors described the upregulation of TLR2, responsible for signaling in response to Gram-positive microbial stimuli, but not of TLR4, on peripheral blood mononuclear cells (PBMCs) of cirrhotic subjects; in addition, TLR2 on PBMCs correlated with circulating levels of both TNF-a and soluble TNF receptors, while TLR4 did not [66].

## 3. Taking a Step Back: Role of MT in the Induction and Progression of Liver Damage in Chronic Viral Hepatitis, Alcohol-Induced Hepatopathy, and NAFLD

So far, we have focused on MT in the context of cirrhosis pathophysiology, but it must be taken into account that MT may work as a “trigger” in the induction and progression of various kinds of hepatic injury (i.e., inflammation, steatosis, fibrosis, and possibly cirrhosis) in several liver diseases, such as alcoholic, metabolic, and viral hepatitis. In fact, the derangement of the gut microflora and MT has been described even at the early stages of liver disease.

**Alcoholic Hepatitis**

Even if alcohol metabolism predominantly occurs in the liver, it is known that colonic bacteria are able to produce large amounts of acetaldehyde from ethanol [67–69]; acetaldehyde is responsible, in turn, of increased intestinal permeability and increased translocation of LPS to the portal vein and on to the liver [70]. In fact, plasma endotoxin levels have been shown to be higher than healthy controls not only in subjects with alcoholic cirrhosis, but also in patients with mild forms of alcoholic hepatitis [71, 72]. In the liver, the LPS/TLR4 pathway has been demonstrated to promote fibrogenesis, by sensitizing stellate cells to transforming growth factor-*β* (TGF-*β*)-induced signals and promoting TGF-*β* release by Kupffer cells [73]. Experiments in rats [74] and clinical studies in humans [75–77] have demonstrated both quantitative and qualitative changes in the gut microflora of alcoholic subjects, in comparison with controls. In addition, ethanol-mediated oxidative stress appears to be an important mechanism that leads to gut leakiness and increased MT [78–81]. In particular, the overproduction of NO and its metabolite peroxynitrite (ONOO-) has been associated with the disruption of the intestinal epithelial layer. NO is synthesized from L-arginine by NOS. Three isoforms of NOS have been identified: neuronal (n)NOS, endothelial (e)NOS, and inducible (i)NOS. While nNOS and eNOS are considered protective for the intestinal mucosa, iNOS may be induced by bacterial products and cytokines and it is believed to significantly contribute to local inflammation and hyperpermeability [82], as confirmed by the observation of reduced alcohol-induced gut leakiness when administering iNOS inhibitors [78]. Furthermore, alcohol has been reported to dysregulate immune responses, suppressing natural killer cell activity, antibody-dependent cell-mediated cytotoxicity, and T-cell-dependent antibody responses, thus globally increasing the host susceptibility to pathogenic bacteria of the gut [83, 84].

**Viral Hepatitis**

Several studies reported elevated LPS levels in the context of chronic viral hepatitis by hepatitis C virus (HCV) and hepatitis B virus (HBV) [85, 86]. In a recent work, Sandler et al. [86] have retrospectively investigated whether the extent and progression of liver disease in patients with chronic HBV or HCV infection were associated with MT and the subsequent local and systemic inflammation. HCV- and HBV-infected individuals had higher plasma levels of LPS, soluble (s)CD14 (produced upon LPS activation of monocytes), and interleukin-6, in comparison with controls. Levels of sCD14 correlated with markers of hepatic inflammation and fibrosis and were significantly higher in subjects with severe fibrosis at presentation, in comparison with those having minimal fibrosis (*P* = 0.01). Importantly, sCD14 was significantly higher in progressors than nonprogressors (*P* = 0.003); moreover, an increase in sCD14 of 1.0×10^6^ pg/ml was associated with an odds ratio of disease progression of 3.7 (*P* = 0.007), with no changes after adjustment for other prognostic factors. Of note, elevated sCD14 levels, but not LPS itself, predicted clinical outcome.

Recent lines evidence coming from studies on HIV-HCV coinfected cohorts have reported a similar correlation between sCD14 and the severity of liver disease [87, 88]. In addition, Marchetti et al. [87] have recently investigated whether baseline MT (as assessed by measuring LPS levels) or host response to MT (sCD14) could be predictive of early virological response (EVR: HCV-RNA <50 IU/mL at week 12 of therapy or ≥2 log10 reduction from baseline after 12 weeks of therapy) and sustained virological response (SVR: HCV-RNA <50 IU/mL 24 weeks after end of therapy) to HCV treatment. In the univariate model, the authors found that lower sCD14 levels were predictive of both EVR and SVR, while LPS were not; however, sCD14 lost its independent predictive value in the multivariable model, thus limiting the possibility to use it in clinical practice as measure to *a priori* include/exclude patients from treatment.

**Metabolic Hepatitis**

Nonalcoholic fatty liver disease (NAFLD) is the most common cause of liver disease in industrialized countries. It usually develops in the setting of obesity and insulin resistance and comprises a continuum of disease ranging from simple steatosis to steatohepatitis (NASH) to cirrhosis. Obesity has been associated with changes in the gut microflora [4] and increased intestinal permeability [89–91]. In subjects with NAFLD, the prevalence of SIBO has also described as higher than controls [91, 92]. A high-fat diet has been reported not only to increase intestinal translocation of endotoxin in mice (the so-called metabolic endotoxemia), but also to reduce enteric *Bifidobacteria* [93, 94], a group of bacteria that have been shown to lower intestinal LPS levels and to improve mucosal barrier function [95]. Furthermore, mice chronically receiving a continuous low rate of LPS, as well as high-fat diet-fed mice, developed insulin resistance, had high circulating levels of proinflammatory cytokines and increased liver triglyceride content. These effects were completely blunted in CD14 mutant mice [94]. Other studies have shown that even the early stages of fructose-induced NAFLD (e.g., steatosis) are associated with increased intestinal translocation of endotoxin and expression of TNF-*α* in the liver, whereas TNF-*α* expression and steatosis were markedly lower in the liver of fructose-fed mice treated with antibiotics or TLR-4-mutant mice [96, 97]. In humans with NAFLD, TNF-*α* expression was reported to be increased too [92, 98]. These data globally support the idea that the LPS/CD14 system has a pivotal role in the induction of the low tone inflammatory status of metabolic disease, setting the threshold of insulin sensitivity and the onset of diabetes and obesity.

## 4. Taking a Step Forward: Role of MT in the Pathogenesis of the Complications of Cirrhosis

The gut microflora has been shown to contribute to the pathogenesis of the complications of cirrhosis, including the development of spontaneous bacterial infections and worsening of the hyperdynamic circulatory state (HCS), a hemodynamic perturbation that may lead to ascites, esophageal variceal growth and hepatorenal syndrome.

Spontaneous bacterial infections (i.e., SBP, empyema, and bacteremia) have an incidence rate of 15%–47% in cirrhotic subjects [99–103] and are mainly caused by Gram-negative bacteria, even though the incidence of infections from Gram-positive cocci has increased in recent series, as a consequence of the use of chronic antibiotic prophylaxis [104–106] and more frequent therapeutic invasive procedures [102]. Mortality is significantly higher in cirrhotic patients developing an infection [101, 103]. SBP, an infection of ascitic fluid typically with a single bacterial species, in the absence of any other primary intra-abdominal source, has been shown to be present in up to 23% of cirrhotic patients with ascites undergoing paracentesis [107]. An increased prevalence of SBP has been described in patients with SIBO [108]; conversely, subjects with SBP have a higher prevalence of SIBO than those without SBP [109]. Animal studies have established a causal link between MT and SBP [14]; in humans, this relationship is supported by the observation that selective intestinal decontamination (SID) by the use of oral nonabsorbable antibiotics is effective in decreasing the incidence of spontaneous bacterial infections in cirrhotic subjects [110–113].

MT has been postulated as an important mechanism in the development of the cirrhotic HCS, which is characterized by low vascular resistance, low mean arterial pressure, and by increased heart rate, cardiac output, and regional blood flow. The HCS is mainly due to splanchnic and systemic vasodilatation, which result in ‘‘underfilling” of the arterial system and subsequent activation of compensatory mechanisms (i.e., renin-angiotensin aldosterone, sympathetic nervous system, and antidiuretic hormone), which are responsible for increased plasma volume and cardiac output. The HCS contributes to portal hypertension and many of the complications of cirrhosis [2, 114]. NO appears to be the key vasodilator responsible for the haemodynamic abnormalities of cirrhosis [115]. It has been reported that gut-derived endotoxins and the resultant increase in inflammatory cytokines are able to induce the expression of iNOS in vessel walls, causing NO overproduction, vasodilatation, and HCS [116]. Endotoxemia has been shown to correlate with serum NO-metabolite levels [117, 118]; moreover, HCS appears to be more marked in patients with cirrhosis and ascites having high levels of LBP [59]. The role of cytokines, specifically TNF-a, in the development of the HCS is evidenced by studies showing that TNF-a inhibitors ameliorate HCS in cirrhotic rats [119]. The importance of the gut microflora in inducing the production of vasoactive mediators has suggested to use SID as a strategy to decrease bacterial overgrowth and MT, in attempts to ameliorate the HCS. Patients treated with a 4-week course of oral norfloxacin demonstrated improvement in biologic and hemodynamic end points, with trends toward reduction in cardiac output, improvements in mean arterial blood pressure and reduced MT [59, 120].

A growing amount of data suggests that bacterial infections may work as an important trigger for variceal bleeding in patients with cirrhosis [121, 122], possibly as a consequence of enhanced activation of hepatic stellate cells, which may increase intrahepatic vascular resistance and portal hypertension and precipitate the coagulopathy that results from prostacyclin-related inhibition of platelet aggregation, consumption of clotting factors by the extrinsic coagulation pathway and reduction of endogenous heparinoids [122–124]. Conversely, variceal haemorrhage predisposes to bacterial infections with gut-derived microflora, thus creating a vicious cycle between gastrointestinal bleeding and infections, which significantly increases the mortality rates in this group of patients [125–127]. 

MT may also participate in the pathogenesis of hepatic encephalopathy (HE). HE is associated with the presence of portal and systemic shunts and the failure of the liver to clear ammonia and other toxic products derived from the gut [128, 129]. Ammonia is produced in both the small bowel (from the effects of glutaminase on glutamine) and large intestine (from urease activity of the colonic flora). Colonic bacteria may also produce gamma-amino-butyric acid (GABA) and benzodiazepine (BZD)-like substances [130, 131], which have been implicated in the development of HE [132, 133]. The role of the gut microflora in the pathogenesis of HE is supported by the observation that total colectomy is effective in reducing baseline and protein-induced ammonia production [134], reversing cases of HE which had not been responsive to medical treatment [135]. Nevertheless, HE may recur, probably as a consequence of the colonization of the small bowel [136].

## 5. Searching for Effective Therapeutic Options: Selective Intestinal Decontamination and Probiotics

Modulation of the intestinal microflora through the use of selective intestinal decontamination (SID) and probiotics has been proposed as an emerging therapeutic strategy in the management of chronic liver diseases.

**SID**

Long-term use of norfloxacin, a poorly absorbed quinolone, has been reported to markedly reduce the count of intestinal aerobic Gram-negative bacilli, but not Gram-positive cocci or anaerobic bacteria [112]. In cirrhotic animals, some studies have shown that the use of oral norfloxacin or trimethoprim/sulfamethoxazole was associated with a decrease in BT [137, 138], while other studies have not [139]. In the clinical setting, a double blind, placebo-controlled trial evaluating the long-term efficacy of norfloxacin in cirrhotic patients who had survived a previous episode of SBP, found a significant reduction of the risk of SBP recurrence in the treated group (20% *versus* 68%) at one year of followup [112]. In cirrhotic patients with low ascitic fluid protein concentrations, long-term prophylactic treatment with norfloxacin was effective even in the prevention of the first episode of SBP, but a major concern of long-term antibiotic prophylaxis is the development of quinolone-resistant infections [140]. Of interest, primary prophylaxis with norfloxacin has been associated with higher rates of trimethoprim–sulfamethoxazole resistance and increased incidence of infections with Gram-positive bacteria, including severe hospital-acquired staphylococcal infections [102, 106, 141]. On the basis of these observations, it is crucial to define non-antibiotic strategies to reduce MT and prevent infections.

**Probiotics**

Probiotics, commonly lactose-fermenting *Lactobacilli *and *Bifidobacteria*, have been reported to stabilize mucosal barrier function and modulate the gut microflora, limiting the growth of pathogenic bacteria, by acidifying the gut lumen, competing for nutrients, and producing antimicrobial substances [95, 142].

*In vitro*, the probiotic *Lactobacillus casei GG* has been shown to inhibit the translocation of *E. coli *in a dose-dependent manner [143]; however, *in vivo* lines of evidence are controversial. Some studies performed in cirrhotic rats have failed to find significant differences in MT rates between cirrhotic animals receiving *Lactobacilli* and untreated controls [144, 145]. On the other hand, Forsyth et al. described reduced gut leakiness and less severe alcoholic steatohepatitis in alcohol plus *Lactobacillus GG*-fed rats, in comparison with alcohol-fed rats [146]. A combination of *Lactobacillus johnsonii LA1* and antioxidants was effective in reducing MT, oxidative damage, and endotoxemia in rats with CCl_4_-induced cirrhosis; unfortunately, the authors did not include a group receiving *Lactobacillus johnsonii LA1* alone [147]. In experimental models of NAFLD, it has been reported that mice treated with VSL#3 (containing *Streptococcus thermophilus, B. breve, B. longum, B. infantis, L. acidophilus, L. plantarum, L. casei,* and* L. bulgaricus*) had lower liver inflammation, serum alanine aminotransferase (ALT), and hepatic total fatty acid content in comparison with controls [148]. Similarly, a recent work of Xu et al. has shown reduced fat accumulation in the liver of high-fat diet-fed rats receiving *Bifidobacterium longum* and in those receiving *Lactobacillus acidophilus*, in comparison with high-fat diet-fed controls. Of interest, intestinal permeability was not affected by probiotics; furthermore, *Bifidobacterium longum* was more effective than *Lactobacillus acidophilus* in attenuating liver fat accumulation [149]. In high-fat diet-fed mice, VSL#3 administration has been associated with decreased hepatic steatosis and insulin resistance, as well as reduced expression of lipid peroxidation markers, TNF-*α*, iNOS, cyclooxygenase 2, and matrix metalloproteinases (MMPs) [150, 151]; in another study, the use of VSL#3 ameliorated hepatic fibrosis, by decreasing expression of procollagen and MMPs, but not steatosis or inflammation [152].

In humans, VSL#3 administration has been reported to reduce oxidative stress in patients with NAFLD and alcoholic liver cirrhosis, but not chronic hepatitis C [153]. Cirrhotic subjects receiving *Escherichia coli Nissle* for 42 days showed a trend toward lower endotoxin levels and improvement in Child-Pugh score [154]; similar results were obtained by Liu et al. when administering a symbiotic compound (a mixture of lactic acid bacteria and fermentable fiber), Synbiotic 2000, to cirrhotic patients [155]. A significant reduction in Child-Pugh class occurred in 47% of patients receiving Synbiotic 2000 for 30 days, whereas this only occurred in 29% of patients randomized to fiber alone and 8% of patients on placebo. Of importance, stool analysis demonstrated the reduction of Gram-negative fecal flora; furthermore, Synbiotic 2000 was associated with a significant improvement of minimal hepatic encephalopathy (MHE) and decreased endotoxemia. On the basis of these observations, it is possible to hypothesize the beneficial role of symbiotics in modulating the gut flora and reducing MT, as confirmed by the lower circulating endotoxin levels found in symbiotic-treated subjects. Opposing results were recently reported in a study of Pereg et al., who failed to show any beneficial effect of probiotic administration in patients with compensated liver cirrhosis [156]. In this double-blind placebo-controlled study, 36 patients were randomly assigned to receive probiotic capsules containing *Lactobacillus acidophilus, Lactobacillus bulgaricus, Bifidobacterium lactis* and* Streptococcus thermophiles* or placebo for 6 months. No differences in either clinical or laboratory parameters between the two groups were found; the small size of the study population, the reduced number of patients having a Child-Pugh class >A, and the longer period of treatment may globally explain the different conclusions of this trial in comparison with those described above.

As refers to the use of probiotics for the prevention of infections, it has been reported that a symbiotic regimen including *Lactobacillus plantarum* and fermentable fiber was more effective than SID in reducing the incidence of bacterial infections in liver transplant recipients [157]. Analogously, a follow-up randomized double-blind trial in liver transplant recipients has shown the rate of postoperative bacterial infections to be significantly lower in subjects receiving a mixture of lactic acid bacteria and fermentable fiber, in comparison with fermentable fiber alone [158].

The effects of the prebiotic lactitol on the intestinal flora and plasma endotoxin levels have recently been evaluated in patients with chronic viral hepatitis [159]. Lactitol and lactulose are synthetic non-absorbable disaccharides, widely used in the treatment of HE; due to the lack of a suitable galactosidase in the upper part of the gastrointestinal tract, they are able to reach undigested the large bowel, where they are metabolized by colonic bacteria, generating organic acids [160]. The resulting lower pH may inhibit urease-producing intestinal bacteria and promote the growth of non-urease-producing lactobacilli [161]; on the basis of these observations, it is unsurprising the report by Chen et al. [159] of increased levels of beneficial bacteria, such as *Bifidobacteria* and *Lactobacilli*, in lactitol-receiving patients with HCV, with a consensual decline in endotoxemia, in comparison with untreated controls.

In the setting of HE, current approaches include the use of non-absorbable antibiotics (i.e. neomycin, paromomycin, metronidazole, or rifaximin) and non-absorbable disaccharides [162]. Probiotics appear to be a promising therapeutic option in the management of HE [155, 163–166]. In fact, some studies have shown that probiotics may positively modulate the gut microflora, reducing the amount of bacterial ammonia reaching the portal vein. The long-term oral administration of *Enterococcus faecium SF 68* was at least as effective as lactulose in improving neurocognitive tests and reducing ammonia levels in patients with HE. Of importance, in this trial the improvement in mental status was maintained during the washout periods in probiotic-treated subjects, but not in lactulose-treated ones [163]. Similarly, cirrhotic patients with minimal hepatic encephalopathy receiving *Bifidobacterium longum* plus fructo-oligosaccharides for 3 months had a significant improvement in both biochemical and neuropsychological tests compared to controls [164].

## 6. Conclusions

The interaction between the gut microflora and the host may play a crucial role in the natural history of chronic liver diseases. In clinical practice, there is a need of surrogate markers of MT and non-antibiotic methods, which may positively modify the gut ecosystem and reduce the passage of bacteria and bacterial products through the gut. Considering the results coming from experimental models and limited clinical studies, probiotics are an attractive strategy, but they need further investigation in larger controlled clinical trials.
